# In Memoriam

**Published:** 1879-05

**Authors:** 


### IN MEMORIAM.

Dr. Geo. B. Wood is no more! On Sunday, March 30th, at his residence on Arch street, Philadelphia, this eminent acologist died, in the eighty-second year of his age.

As a teacher and writer few have equalled, and none have excelled him in his favorite department of Medical Science, in Europe or America. The *United States Dispensatory*, two-thirds of which was written by him, is the most extensive and critically correct history of remedies, their reputed action and application in the treatment of disease, that was ever written.

Peace to the remains of this eminent man!

